# Recent Advances in Ultrasound-Guided Hydrodissection and Dextrose Prolotherapy for Musculoskeletal Pain: A Target-Based State-of-the-Art Narrative Review

**DOI:** 10.3390/life16091438

**Published:** 2026-08-28

**Authors:** Yonghyun Yoon, Rowook Park, Jaehyun Shim, Seungbeom Kim, Jonghyeok Lee, Liza Maniquis-Smigel, King Hei Stanley Lam, Teinny Suryadi, Anwar Suhaimi

**Affiliations:** 1 Department of Orthopaedic Surgery, Kangnam Sacred Heart Hospital, College of Medicine, Hallym University, 1 Singil-ro, Yeongdeungpo-gu, Seoul 07441, Republic of Korea; 2 Incheon Terminal Orthopedic Surgery Clinic, Inha-ro 489beon-gil, Namdong-gu, Incheon 21574, Republic of Korea; 3 International Academy of Regenerative Medicine, Inha-ro 489beon-gil, Namdong-gu, Incheon 21574, Republic of Korea; 4 The Board of Clinical Research, The International Association of Musculoskeletal Medicine, Kowloon, Hong Kong, China; 5 Department of Rehabilitation Medicine, Sae Yonsei Rehabilitation Clinic, Seoul 03186, Republic of Korea; 6 Department of Neurosurgery, Chungdammadi Neurosurgery Clinic, Seoul 03186, Republic of Korea; 7 Miso Pain Clinic, 1569, Bongyeong-ro, Yeongtong-gu, Suwon-si 16703, Republic of Korea; 8 Bareun Neurosurgery Clinic, 39, Daenong-ro, Heungdeok-gu, Cheongju-si 28402, Republic of Korea; 9 Private Practice PM&R and Pain Management, Hilo, HI 96720, USA; 10 Faculty of Medicine, The University of Hong Kong, Hong Kong, China; 11 Faculty of Medicine, The Chinese University of Hong Kong, Hong Kong, China; 12 Department of Physical Medicine and Rehabilitation, Medistra Hospital, South Jakarta 12950, Indonesia; 13 Department of Physical Medicine and Rehabilitation, Synergy Clinic, West Jakarta 11510, Indonesia; 14 Department of Rehabilitation Medicine, Universiti Malaya, Kuala Lumpur 50603, Malaysia

**Keywords:** hydrodissection, dextrose prolotherapy, ultrasound-guided injection, peripheral nerve entrapment, tendinopathy, connective tissue, musculoskeletal pain

## Abstract

Ultrasound-guided hydrodissection and dextrose prolotherapy have evolved into anatomically explicit but mechanistically distinct strategies for musculoskeletal pain. This State-of-the-Art narrative review aimed to synthesize major recent clinical and technical developments and to interpret them within a target-based framework. PubMed and Embase via Ovid were searched for English-language publications from 1 January 2021 through 29 July 2026, supplemented by selected pre-2021 landmark studies identified through backward citation tracking. After initial deduplication, 1662 records entered Title/Abstract screening. The final narrative synthesis cited 67 publications, including selected landmark and methodological sources. One author performed screening, and a second author reviewed the final publication selection; study selection was purposive and designed for a narrative synthesis rather than exhaustive systematic inclusion. Four questions guided the review: recent refinements in hydrodissection technique and indications; increasing condition specificity of prolotherapy evidence; areas of overlap and distinction between the two procedures; and the evidentiary status of emerging applications. Hydrodissection research remains concentrated in carpal tunnel syndrome and increasingly addresses injectate, volume, tissue-plane spread, and procedural configuration, whereas evidence beyond the carpal tunnel remains incompletely replicated. Prolotherapy research has shifted from broad claims toward condition-specific studies in tendinopathy, plantar fasciopathy, knee osteoarthritis, periarticular disorders, and temporomandibular joint pathology. Across both fields, variations in concentration, volume, anatomical site, session number, comparator, rehabilitation, and procedural expertise limit generalization. Hydrodissection is defined by observable, fluid-mediated separation of a tissue interface, whereas prolotherapy involves dextrose delivery to a symptomatic connective-tissue target without requiring immediate tissue-plane separation. The proposed framework is intended to standardize terminology, organize evidence maturity, and guide future comparative research rather than prescribe a validated treatment algorithm.

## 1. Introduction

Musculoskeletal conditions are major contributors to disability and rehabilitation needs worldwide [1]. In clinical practice, regional pain may arise from neural, fascial, tendinous, ligamentous, capsular, or enthesis-related structures rather than from a single tissue diagnosis. Real-time ultrasonography enables dynamic visualization of these structures, needle guidance along a planned trajectory, and direct observation of injectate distribution [2].

Ultrasound-guided hydrodissection and dextrose prolotherapy are increasingly used in nonoperative musculoskeletal practice, but their terminology remains inconsistent. Hydrodissection has been described using the injectate, the regional diagnosis, or the target nerve, even though its defining procedural feature is fluid-mediated separation of a tissue interface [3,4,5,6]. Prolotherapy is likewise frequently reduced to the label “dextrose injection” despite substantial differences in dextrose concentration, anatomical target, injection pattern, and rehabilitation context [7,8,9,10].

The distinction is clinically important because 5% dextrose may be used during perineural hydrodissection, where visible separation or circumferential spread is the intended procedural endpoint, whereas hypertonic dextrose is commonly used in prolotherapy directed at tendons, ligaments, entheses, capsules, or periarticular tissues [3,5,8,10,11]. In this review, hydrodissection is defined by fluid-mediated separation of a specified tissue interface, whereas dextrose prolotherapy is defined by target-directed dextrose delivery to a connective-tissue or periarticular structure without requiring tissue-plane separation. For terminology within this review, dextrose concentrations above 5% are described as hypertonic; concentration alone does not define the intervention. These procedures may share an injectate but do not necessarily share the same therapeutic logic.

During the past five years, the literature has changed in two important ways. First, hydrodissection research—particularly in carpal tunnel syndrome—has moved beyond feasibility toward comparative questions involving injectate, volume, comparator, and durability [12,13,14,15,16,17,18,19,20,21]. Second, prolotherapy research has become increasingly condition-specific, with systematic reviews and controlled trials examining discrete tendinopathies, plantar fasciopathy, knee osteoarthritis, and temporomandibular joint disorders [22,23,24,25,26,27,28,29,30,31].

This review had two overarching objectives: (1) to synthesize recent clinical and technical advances in ultrasound-guided hydrodissection and dextrose prolotherapy; and (2) to organize those advances within a target-based framework that separates evidence-supported practice from anatomical feasibility and hypothesis-generating applications. These objectives were operationalized through four questions concerning hydrodissection technique and indications, the increasing condition-specific prolotherapy literature, the shared and distinct features of the procedures, and the evidentiary status of emerging applications. The framework is intended to improve terminology, reporting, and study design rather than prescribe a validated sequence of treatment.

## 2. Review Methodology and Conceptual Framework

### 2.1. Review Design and Questions

This article was designed as a State-of-the-Art narrative review with a focused update of the recent literature and a conceptual synthesis. The manuscript was structured using the principles underlying the SANRA framework, including a clear statement of purpose, transparent description of the literature identification, appropriate referencing, scientific reasoning, and presentation of clinically relevant data [32].

Consistent with the two objectives stated above, the review addressed four review questions: (1) What recent evidence has refined the technique and indications of ultrasound-guided hydrodissection? (2) How has the evidence for dextrose prolotherapy become more condition- and site-specific? (3) Which concepts are shared by the two procedures, and which should remain distinct? (4) Which emerging applications have only anatomical, technical, or case-based support?

### 2.2. The Focused Literature Identification

PubMed and Embase via Ovid (accessed on 29 July 2026) were searched for English-language publications from 1 January 2021 through 29 July 2026. Three search domains were used in each database: hydrodissection, dextrose prolotherapy, and perineural dextrose/mechanistic overlap. The searches retrieved 635 PubMed and 1411 Embase records. After within-database duplicate handling and application of the English-language limit, 590 PubMed and 1194 Embase records remained; initial cross-database deduplication yielded 1662 records for Title/Abstract screening. One author (Y.Y.) performed Title/Abstract screening, and a second author reviewed the final publication selection. Full database-specific search strategies and limits are provided in Supplementary Table S1.

The search was designed to identify representative and methodologically informative publications rather than to achieve exhaustive inclusion of every potentially eligible report. Conference abstracts/records, study protocols without completed outcome data, non-musculoskeletal hydrodissection, systemic glucose studies without a relevant local injection intervention, and clearly out-of-scope publications were excluded during screening. Priority was given to systematic reviews, meta-analyses, randomized or comparative clinical studies, prospective cohorts, and studies that materially advanced understanding of injectate selection, volume, concentration, anatomical target, procedural endpoint, patient selection, or safety. Backward citation tracking identified selected pre-2021 landmark publications required for terminology, mechanisms, and historical context. Because this was a State-of-the-Art narrative review, final publication selection was purposive and guided by relevance to the review questions and methodological informativeness rather than by a PRISMA-style exhaustive inclusion process. The final manuscript cites 67 publications, including recent database-identified studies, methodological and contextual references, and selected pre-2021 landmark publications.

### 2.3. Evidence Prioritization and Interpretation

Evidence was organized by intervention, anatomical target, study design, and independent replication. Systematic reviews and randomized or comparative studies were prioritized when discussing clinical effectiveness. Observational studies were included when they addressed populations or procedural questions not represented in controlled trials. Cadaveric and technical studies were included only when they introduced a clinically relevant target or validated an injection pathway. Case reports were used selectively to illustrate emerging applications and were not interpreted as evidence of efficacy. Study-level characteristics of the principal comparative clinical studies informing the synthesis are summarized in Supplementary Table S2.

No formal risk-of-bias instrument, certainty-of-evidence grading, or quantitative pooling was performed because the review was not designed as a systematic review or meta-analysis. For descriptive purposes, evidence maturity was categorized according to study design and independent replication as replicated comparative evidence (comparative findings independently replicated or synthesized across studies), limited comparative evidence (comparative data without consistent independent replication), observational/feasibility evidence (noncomparative clinical or feasibility studies), or anatomical/technical/case-based evidence (cadaveric, technical, or case-level reports without comparative clinical validation). These categories were used solely to organize the literature and do not represent a formal certainty-of-evidence assessment.

### 2.4. Operational Definitions

The following operational definitions were used to reduce conceptual ambiguity. Hydrodissection refers to an ultrasound-guided procedure in which fluid is used to visibly separate a target structure from an adjacent tissue plane. Perineural injection refers more broadly to injectate placement around a nerve and should not automatically be labeled hydrodissection unless separation is demonstrated. Interface restriction denotes a clinically suspected reduction in relative mobility between adjacent tissues; no validated universal sonographic threshold currently exists. Tissue gliding refers to dynamic relative movement observed during a standardized maneuver. Dextrose prolotherapy refers to dextrose delivery to a selected connective-tissue or periarticular target with an intended biologic and/or neuromodulatory effect. For terminology in this review, dextrose concentrations above 5% are described as hypertonic; concentration alone does not define prolotherapy. A suspected symptomatic structure or interface denotes the anatomical target considered most relevant after integration of history, examination, imaging, dynamic assessment, and, when appropriate, electrodiagnostic or diagnostic procedural information; this inference does not establish causality.

### 2.5. Ultrasound Guidance, Operator Expertise, and Procedural Reproducibility

Ultrasound guidance is part of the intervention rather than a localization step alone. Transducer selection should match target depth and field of view: high-frequency linear transducers are generally suited to superficial nerves, tendons, entheses, and fascial interfaces, whereas deeper or curved regions may require a lower-frequency linear or curvilinear transducer. A bulky probe footprint can restrict the needle-entry window in anatomically confined regions [2].

Procedural reproducibility is operator-dependent. Probe orientation, beam–needle angle, needle-tip visualization, recognition of anisotropy and adjacent neurovascular structures, and interpretation of injectate spread may influence procedural fidelity. Probe and palpation pressure are additional sources of variability because excessive compression may alter tissue geometry, nerve caliber, vascular appearance, and apparent tissue mobility, whereas inconsistently applied pressure may affect symptom reproduction and dynamic assessment. Sonoguided digital palpation (SDP) is an emerging dynamic sono-clinical assessment in which controlled digital pressure is applied while the target structure is visualized in real time. In a recent report involving the long thoracic nerve, SDP reproduced concordant pain by targeted palpation of the visualized nerve before ultrasound-guided hydrodissection [33]. However, the diagnostic validity, pressure standardization, interobserver reliability, and clinically meaningful thresholds of SDP remain unestablished; it should therefore be regarded as a hypothesis-generating adjunct rather than a validated diagnostic test. For hydrodissection, technical success should document the intended tissue-plane opening, circumferential spread, or comparable real-time evidence of separation rather than perineural fluid placement alone. The hydraulic force generated during injection may also vary with needle position, tissue compliance, injection rate, and volume, but no validated injection-pressure threshold currently defines adequate or excessive tissue-plane separation [2,3,6].

Local expertise is consequently relevant when translating procedural evidence into practice. Future studies should report on the number of operators, relevant ultrasound-intervention training or experience, and any protocol used to standardize technique across operators or centers. Where pressure-based dynamic assessment or hydrodissection is used, reporting should also describe the palpation or transducer-pressure maneuver, injection rate or perceived resistance where feasible, and the sonographic endpoint used to confirm tissue-plane separation. Such reporting may improve procedural reproducibility and interpretation across operators and centers.

## 3. Conceptual and Terminological Foundations

**Figure 1:**
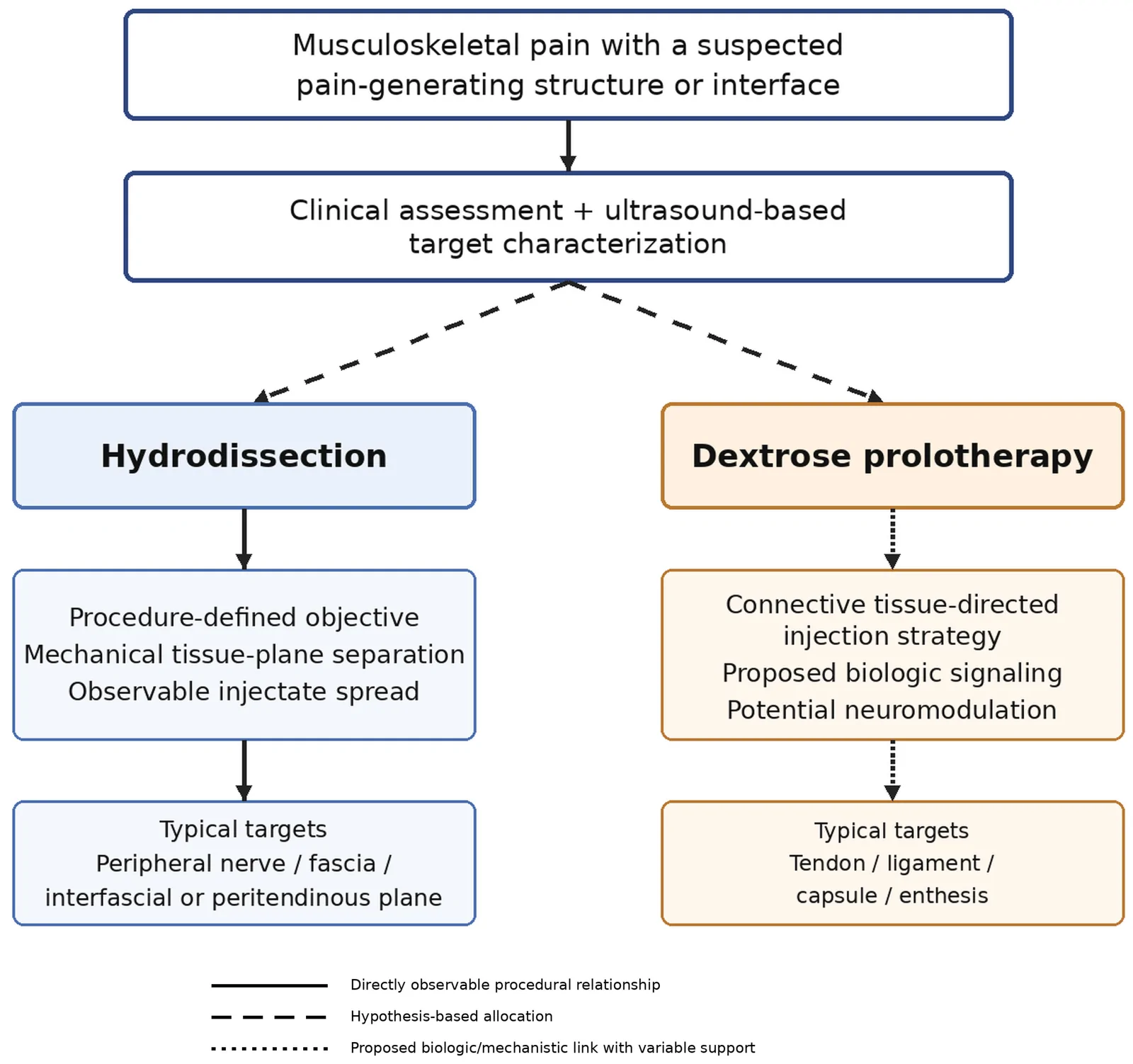
Target-based conceptual framework. Solid arrows indicate directly observable procedural relationships; long-dashed arrows indicate hypothesis-based allocation from clinical assessment to an intervention; dotted arrows indicate proposed biologic or mechanistic links with variable support. The model is intended for reasoning and research design and is not a validated treatment algorithm.

The same injectate can be used for different procedural objectives, and the same regional diagnosis can arise from different target tissues. A target-based framework therefore starts with the structure or interface being treated, the expected procedural endpoint, and the maturity of supporting evidence rather than with the injectate alone (Table 1 and Figure 1).

## 4. Recent Advances in Ultrasound-Guided Hydrodissection

### 4.1. Consolidation of Evidence in Carpal Tunnel Syndrome

Carpal tunnel syndrome (CTS) remains the best-developed clinical model for nerve hydrodissection. Earlier reviews established technical feasibility and an acceptable safety profile but also identified uncertainty regarding the active component of treatment, the preferred injectate, volume, approach, and frequency [4,12]. More recent systematic reviews have confirmed that most controlled hydrodissection evidence remains concentrated in CTS [13,14].

A 2025 network meta-analysis of nine studies involving 458 patients ranked 5% dextrose highly for several short- and intermediate-term symptom and function outcomes. However, indirect comparisons, limited trial numbers, and variation in technique prevent the rankings from being treated as definitive protocol recommendations [15]. The central development is therefore not proof that one universal injectate has been established, but recognition that injectate and technique are likely treatment-effect modifiers.

### 4.2. Injectate, Volume, and Separation Endpoint

Randomized trials have compared 5% dextrose in water (D5W) with saline or corticosteroid and have separately examined volume-dependent effects [16,17,18,19,20]. In a four-arm double-blind trial, 10 mL D5W produced greater short-term improvement in pain and median nerve cross-sectional area than the saline groups, whereas a separate saline-based volume study compared 5- and 10-mL hydrodissection without establishing a universally optimal volume [16,17]. These findings support the plausibility of both volume-dependent mechanical separation and injectate-specific effects, but they do not isolate the relative contribution of either mechanism. In the saline-volume trial, the 10-mL group showed greater improvement than the 5-mL group at 24 weeks in BCTQ symptom severity (median change, −0.8 vs. −0.5; *p* = 0.024) and functional status (mean change, −0.8 vs. −0.5; *p* = 0.011) [17].

Comparisons with corticosteroids further illustrate the classification problem. A perineural injection may be accurately placed without producing a substantial hydrodissection endpoint, whereas a larger-volume injection may visibly separate the median nerve from the flexor retinaculum and subsynovial connective tissue. Trials comparing D5W and corticosteroid, and hydrodissection with or without corticosteroid, suggest that both injectate and procedural configuration influence outcomes [18,19,20]. Accordingly, future reports should document the plane approached, volume, spread pattern, and whether separation was achieved.

### 4.3. Dynamic Ultrasound and Procedural Phenotyping

Potential ultrasound-observable procedural endpoints include circumferential perineural spread, opening of a restricted fascial plane, change in nerve excursion, and alteration in cross-sectional area [2,3,6]. These observations link the intervention to an immediate anatomical event, but none has been validated as a surrogate endpoint for clinical benefit.

A single-entry dual-target technique with cadaveric validation illustrates the trend toward anatomically explicit procedural design [34]. Its value lies in demonstrating a reproducible pathway and distribution pattern; clinical superiority over conventional approaches remains unproven. This distinction—technical reproducibility versus therapeutic effectiveness—should be maintained throughout the literature.

### 4.4. Expansion Beyond Primary Carpal Tunnel Syndrome

Recent studies have extended dextrose-based perineural treatment beyond primary CTS. Randomized evidence has been reported for meralgia paresthetica and ulnar neuropathy at the elbow [35,36], while an observational series has described persistent or recurrent CTS after surgery [21]. The meralgia paresthetica study reported ultrasound-guided hydrodissection, whereas the ulnar neuropathy study is more appropriately described as ultrasound-guided perineural dextrose injection because tissue-plane separation was not established as the defining procedural endpoint [35,36]. Because only two non-carpal entrapment sites and one postsurgical phenotype have been evaluated in comparative or observational studies, extension to all entrapment neuropathies would be premature.

Interfascial and myofascial hydrodissection has also been evaluated in randomized trials involving shoulder-neck dysfunction and upper trapezius myofascial pain [37,38]. This work broadens the procedural focus from perineural decompression to mobility between fascial layers. Replication should use prespecified dynamic ultrasound measures because diagnostic thresholds for fascial restriction remain undefined.

A randomized comparison of hydrodissection and open surgery in severe CTS is notable because it places a minimally invasive procedure against a definitive decompressive treatment [39]. The result should be interpreted cautiously: surgical candidacy, electrodiagnostic severity, symptom duration, and follow-up length are critical modifiers, and a single trial cannot establish equivalence to surgery.

### 4.5. Anatomical and Case-Based Innovation

Cadaveric and anatomical investigations can validate needle pathways, target access, and dye distribution. A deep-gluteal fascial-plane study demonstrated access to the external-rotator enthesis region and the vicinity of the sciatic nerve, providing an anatomical basis for a mixed interface–enthesis hypothesis [40]. Such work is useful for procedural development but cannot reproduce in vivo pressure, fascial compliance, neural excursion, or clinical response.

Representative case reports have described dextrose hydrodissection for Wartenberg syndrome, sural nerve entrapment in postoperative scar, and subclavius-region pathology in neurogenic thoracic outlet syndrome [41,42,43]. Beyond illustrating anatomical localization and procedural reasoning, these reports provide no reliable estimate of response rate, durability, comparative effectiveness, or generalizability. All three originated from overlapping author networks represented in this review; independent prospective validation is therefore essential. Recent hydrodissection developments and unresolved questions are summarized in Table 2.

## 5. Recent Advances in Dextrose Prolotherapy

### 5.1. From Broad Indications to Condition-Specific Evidence

Foundational reviews described dextrose prolotherapy as a broad connective-tissue injection strategy and emphasized non-uniform protocols and uncertain mechanisms [7,8,9,10]. The recent literature is more informative when organized by anatomical site and clinical condition rather than by the generic label “prolotherapy”.

A 2024 systematic review of randomized trials in sports-related tendinopathies found generally favorable signals but substantial variation in tendon, concentration, comparator, injection schedule, and rehabilitation [22]. This pattern is consistent with condition-specific reviews in lateral elbow tendinosis and rotator cuff tendinopathy [23,24]. The evidence supports continued condition-specific investigation but does not support a uniform protocol or generalized recommendation across all tendon disorders.

### 5.2. Rotator Cuff, Lateral Elbow, and Plantar Fasciopathy

Rotator cuff and supraspinatus studies illustrate the importance of anatomical specification. A systematic review and randomized trials have reported potential improvement after hypertonic dextrose injections, with injection sites ranging from intratendinous and enthesis-directed placement to bursal and periarticular approaches [24,25,44]. The term “rotator cuff prolotherapy” therefore conceals clinically relevant variation in technique. In the 2022 randomized trial, the dextrose group showed short-term within-group improvement at 2 weeks in VAS (mean difference, −2.1; 95% CI, −2.7 to −1.4) and SPADI (mean difference, −11.6; 95% CI, −16.5 to −6.7), although these effects were not sustained to 6 weeks [25].

Plantar fasciopathy has one of the larger condition-specific prolotherapy literature. Recent meta-analyses of randomized trials report potential improvements in pain and function, while comparisons with corticosteroid and extracorporeal shock-wave therapy show that relative benefit depends on follow-up interval and outcome selection [26,27,45,46,47]. The early sonographically guided pilot literature remains historically important because it established anatomically guided injection, although it should not carry the same evidentiary weight as later comparative trials [48].

For lateral epicondylitis, systematic review evidence and randomized trials indicate potential benefit [23,49,50]. In the direct comparison of low- and high-dose dextrose, the higher concentration did not demonstrate a clear clinical advantage [49]. A recent multiarm trial with two-year follow-up compared physiotherapy, shock-wave therapy, prolotherapy, and platelet-rich plasma, highlighting the need to interpret prolotherapy within a broader rehabilitation and orthobiologic landscape rather than as an isolated intervention [51].

### 5.3. Knee Osteoarthritis and Periarticular Phenotyping

In knee osteoarthritis, meta-analyses of hypertonic dextrose prolotherapy report favorable pooled estimates for some pain and functional outcomes, although protocol variation and imprecision reduce certainty [28,29].

Recent trials have examined dextrose concentration, combined intra-articular and periarticular/perineural treatment, and comparison with intra-articular saline [52,53,54]. By varying concentration, injection compartment, and the addition of periarticular perineural treatment, these trials test clinically relevant protocol components rather than a generic treatment effect. The available designs do not determine whether improvements arise predominantly from intra-articular, periarticular, perineural, or multimodal mechanisms.

Site-specific studies in pes anserine bursitis and subacromial bursitis further demonstrate that localized periarticular disorders should not be merged automatically with intra-articular osteoarthritis or tendinopathy [55,56]. The diagnosis, target tissue, injectate concentration, and co-intervention should be explicit.

### 5.4. Capsular, Enthesis-Related, and Temporomandibular Targets

Capsular and enthesis-related applications remain heterogeneous. A randomized frozen-shoulder study incorporated molecular markers related to matrix remodeling, while a prospective study of acromial enthesopathy and acromioclavicular arthropathy used ultrasound-guided target localization [57,58]. Molecular and ultrasound-based characterization adds biological and anatomical context. A reproducible capsuloligamentous protocol has nevertheless not emerged because site definitions, dextrose concentration, injection pattern, and outcome assessment remain non-uniform.

Temporomandibular joint (TMJ) prolotherapy has accumulated systematic reviews, randomized comparisons, and pilot studies [30,31,59,60,61,62]. The literature has progressed from proof-of-concept toward comparisons of ultrasound guidance, dry needling, autologous conditioned serum, and defined internal-derangement phenotypes. Diagnostic classification, injection compartment, concentration, and treatment frequency nevertheless remain non-uniform.

### 5.5. Anatomical Target Development and Representative Cases

Recent cadaveric and technical studies have proposed ultrasound-guided access to the anterior labral–ligamentous complex and popliteal fascial-retinacular structures [63,64]. Their scientific contribution is anatomical validation and procedural reproducibility; they do not demonstrate that dextrose improves clinical outcomes at these targets.

Representative case reports have described piriformis enthesopathy, sacrospinous-ligament calcification, and A1-pulley calcification treated with dextrose prolotherapy [65,66,67]. Their appropriate role is to define candidate structural phenotypes and research questions, not to establish efficacy. These publications also arise from overlapping collaborative networks; replication by independent groups is required before clinical expansion. Table 3 summarizes the principal prolotherapy evidence domains and their limitations.

## 6. Convergence and Divergence of the Two Approaches

Hydrodissection and dextrose prolotherapy converge in three areas. First, both benefit from precise ultrasound-based target characterization. Second, dextrose may contribute neuromodulatory effects in perineural treatment and may also be used at connective-tissue targets [5,8,10,11]. Third, complex regional pain may involve simultaneous neural-interface and connective-tissue pathology.

The practical consequence is that identical injectates do not create identical interventions. A hydrodissection procedure is verified during injection by opening or filling the intended tissue plane. In prolotherapy, technical success concerns accurate deposition at a predefined tendon, ligament, capsule, enthesis, or periarticular site; no immediate separation endpoint is required. This difference determines what should be reported and prevents the label “dextrose injection” from conflating distinct procedures.

**Figure 2:**
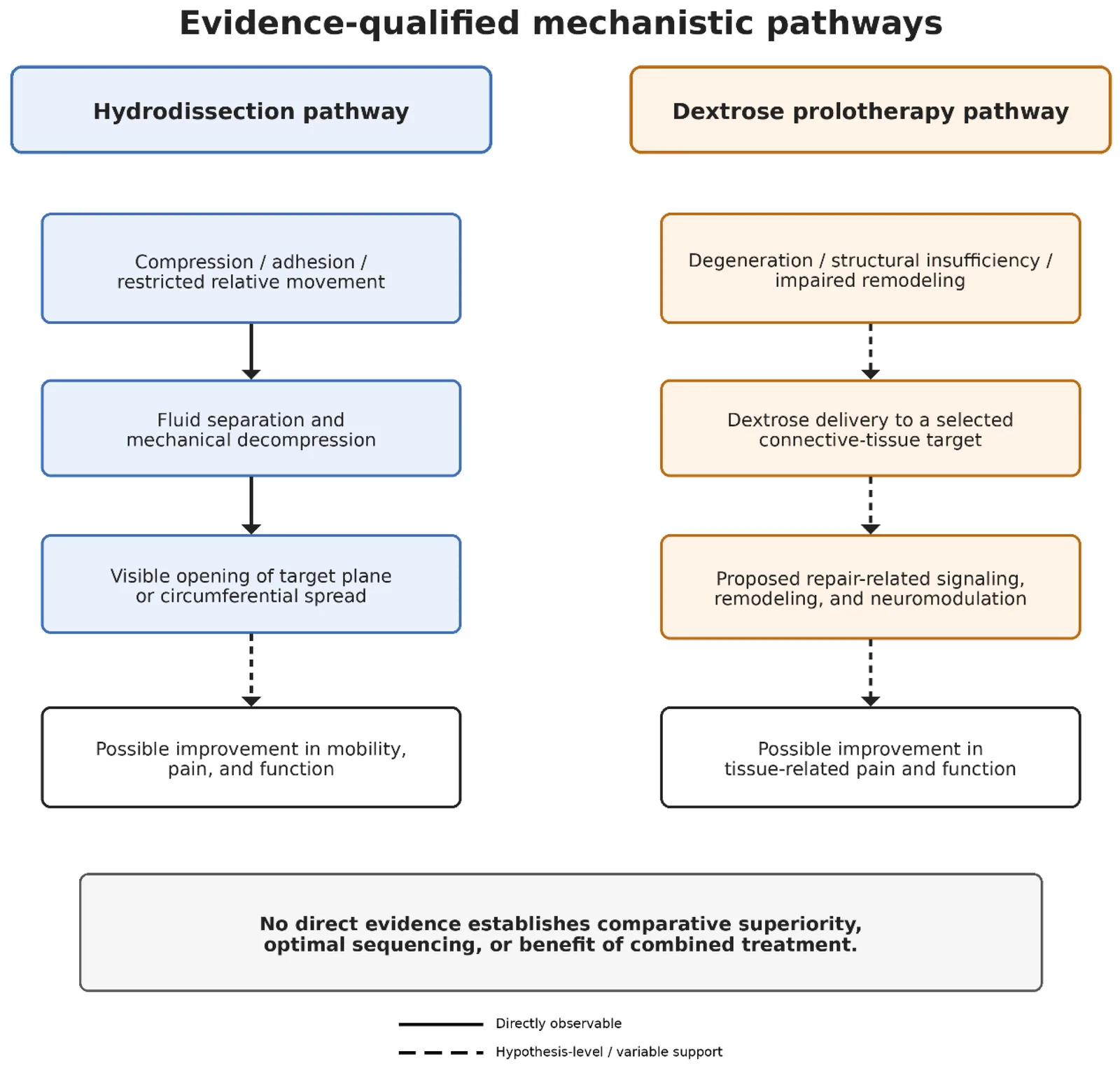
Evidence-qualified mechanistic pathways. Solid arrows indicate directly observable procedural events; dashed arrows indicate proposed or variably supported mechanistic links. No direct evidence establishes comparative superiority, optimal sequencing, or benefit of combined treatment.

Direct comparative evidence between a hydrodissection-first strategy and a prolotherapy-first strategy is absent. Likewise, there is insufficient evidence that combining or sequencing the two procedures improves outcomes. In selected mixed presentations, staged treatment represents an individualized hypothesis followed by reassessment rather than an evidence-based algorithm. Figure 2 distinguishes directly observable procedural events from downstream links that remain mechanistic hypotheses.

## 7. Proposed Target-Based Interpretive Framework

### 7.1. Establish a Testable Structural Hypothesis

The framework begins with a testable hypothesis about the symptomatic structure or interface rather than an assumption that a regional diagnosis identifies a single pain generator. History, physical examination, focal symptom reproduction, ultrasound morphology, dynamic movement, and relevant electrodiagnostic or cross-sectional imaging should be integrated. A sonographic abnormality alone does not establish causality.

Hydrodissection is most coherent when the hypothesis involves nerve compression, perineural adhesion, scar tethering, or a restricted fascial plane and when the intended endpoint can be observed. Dextrose prolotherapy is most coherent when symptoms localize to a tendon, ligament, enthesis, capsule, or periarticular connective tissue and the target can be described anatomically. These statements are intended as classification principles for research and reporting, not as validated diagnostic thresholds or treatment recommendations.

### 7.2. Match the Procedure to the Endpoint

For hydrodissection, the report should specify the structure separated, needle approach, injectate, concentration, volume, distribution pattern, and dynamic or morphological endpoint. For prolotherapy, the report should specify the exact target, concentration, volume, number of injection points, session interval, local anesthetic use, and rehabilitation protocol.

Mixed or uncertain presentations should prompt staged reassessment rather than automatic multi-target injection. Improvement after a procedure supports—but does not prove—the original pain-generator hypothesis because natural history, contextual effects, rehabilitation, and nonspecific needle or injectate effects may contribute.

## 8. Research Agenda and Reporting Priorities

The recent literature supports a shift from broad intervention labels toward reproducible phenotyping and procedural reporting. Priority questions are summarized in Table 4. Safety reporting also requires greater standardization. Reported adverse events are commonly transient, including procedural pain or soreness, localized bruising or swelling, and short-lived sensory symptoms; uncommon complications may include inadvertent intraneural or intravascular injection, bleeding, infection, or injury to adjacent structures. The absence of serious events in small studies should not be interpreted as evidence that rare complications do not occur; target, needle approach, injectate, concentration, volume, monitoring period, and adverse-event definitions should be reported explicitly.

## 9. Discussion

The four review questions yielded four principal conclusions. First, hydrodissection research has progressed from feasibility toward protocol optimization, although carpal tunnel syndrome remains the only indication with a substantial comparative evidence base. Second, prolotherapy studies are increasingly condition-specific and anatomically explicit, but no uniform concentration, injection pattern, or rehabilitation strategy has emerged. Third, the procedures share ultrasound-guided anatomical precision and may both employ dextrose, yet their technical endpoints and evidentiary claims should remain distinct. Fourth, emerging applications are supported mainly by anatomical, technical, or case-based reports and therefore require independent validation before clinical expansion. Collectively, both fields have moved from broad efficacy claims toward questions of dose, distribution, procedural endpoint, and patient phenotype.

The target-based framework offers two scientific advantages. First, it separates a directly observable procedure—fluid-mediated tissue-plane separation—from proposed downstream mechanisms. Second, it prevents the term “dextrose injection” from collapsing perineural hydrodissection and connective-tissue prolotherapy into a single intervention. This distinction should improve comparability among future studies.

The framework also imposes limits. A suspected symptomatic structure or interface is usually inferred rather than proven. Dynamic ultrasound findings are operator-dependent and lack validated thresholds for many interfaces. Operator training, procedural experience, equipment, and local technical capability may influence target identification, needle visualization, injectate distribution, and reproducibility. Clinical responses cannot uniquely identify the mechanism. For these reasons, the framework should be used to formulate and test hypotheses, not to justify unvalidated multi-target treatment.

Recent anatomical and case-based innovation is valuable but particularly vulnerable to confirmation bias, selective reporting, and premature clinical expansion. The appropriate translational sequence is anatomical validation, technical reproducibility, early safety assessment, prospective clinical evaluation, and independent comparative replication.

## 10. Limitations

This is a focused narrative review rather than a systematic review or meta-analysis. The literature search was structured and is reported in Supplementary Table S1, but publication selection was purposive rather than exhaustive; no protocol was prospectively registered, and no formal risk-of-bias or certainty-of-evidence instrument was applied. Restriction to English-language publications and two bibliographic databases may have omitted relevant studies. Title/Abstract screening was performed by one author with final publication selection reviewed by a second author; the absence of duplicate independent screening and the use of backward citation tracking and purposive inclusion may introduce selection or citation bias.

The evidence varies substantially in diagnosis, anatomical definition, injectate composition, concentration, volume, injection technique, comparator, rehabilitation, follow-up, and outcome selection. Several trials are small or single-center, and many non-carpal or anatomically novel applications remain supported by technical reports, cadaveric studies, or case reports.

Some emerging publications were authored by overlapping collaborative networks that include authors of this review. To reduce overinterpretation, these reports were used only to illustrate anatomical feasibility or generate hypotheses, and the need for independent validation has been emphasized. Non-financial professional involvement in teaching ultrasound-guided procedures is disclosed below.

## 11. Conclusions

Over the past five years, both fields have moved toward anatomically explicit protocols and more cautious, condition-specific interpretation. For hydrodissection, the most mature evidence remains carpal tunnel syndrome, while volume, injectate, and procedural endpoints require further study. For dextrose prolotherapy, evidence is broader across connective-tissue disorders but remains limited by non-uniform protocols and rehabilitation. The proposed framework separates an observable mechanical intervention from a connective-tissue injection strategy and categorizes emerging applications according to evidence type and replication. Its translational value lies in improving patient phenotyping, procedural reporting, and trial design, not in prescribing a fixed sequence of care. Independent comparative studies should test whether this classification improves selection, safety, and durable outcomes.

## Figures and Tables

**Table 1:** Operational distinction between hydrodissection and dextrose prolotherapy.

Domain	Hydrodissection	Dextrose Prolotherapy	Interpretive Caution
Defining feature	Visible separation of a nerve, fascia, tendon sheath, or interfascial plane	Dextrose delivery to a symptomatic tendon, ligament, enthesis, capsule, or periarticular target	The injectate alone does not define the procedure
Common dextrose formulation	5% dextrose is frequently used in perineural practice	Hypertonic dextrose (>5%) is common, but concentration varies by indication	Concentration and procedural purpose should be reported separately
Primary procedural endpoint	Opening of a target plane, circumferential spread, or an observed change in relative mobility	Accurate deposition at a predefined connective-tissue target	Pain relief alone does not verify that the intended mechanism occurred
Best-developed evidence	Carpal tunnel syndrome	Selected tendinopathies and plantar fasciopathy	Evidence should not be generalized automatically to novel targets
Emerging applications	Non-carpal neuropathies, postoperative scars, interfascial and myofascial planes	Capsular, enthesis-related, periarticular, and anatomically novel targets	Cadaveric feasibility and case reports do not establish effectiveness

Note: Supporting references are cited in the corresponding Sections in the main text.

**Table 2:** Major recent developments in ultrasound-guided hydrodissection.

Domain	Representative Recent Evidence	Advance	Current Interpretation	Unresolved Issue
CTS evidence synthesis	Systematic reviews and network meta-analysis	Consolidation of short- and intermediate-term efficacy data	Replicated comparative evidence in selected CTS outcomes	Long-term durability and external validity
Volume and injectate	Randomized D5W, saline, and corticosteroid comparisons	Recognition of volume and injectate as separate variables	No single protocol is definitive	Mechanical versus injectate-specific contribution
Postoperative CTS	Retrospective D5W series	Reported postoperative application	Observational evidence; uncontrolled	Prospective comparator and surgical phenotype
Non-carpal neuropathy	Meralgia and ulnar neuropathy trials	Expansion beyond median nerve	Condition-specific evidence only	Independent replication and site-specific dosing
Fascial/myofascial interfaces	Randomized interfascial studies	Extension beyond classic entrapment neuropathy	Limited comparative evidence	Validated diagnostic and dynamic endpoints
Anatomical innovation	Cadaveric and technical studies	Reproducible access and distribution mapping	Anatomical/technical evidence	Clinical effectiveness and safety

Note: CTS, carpal tunnel syndrome; D5W, 5% dextrose in water. Supporting references are cited in the corresponding Sections in the main text.

**Table 3:** Recent evidence domains in dextrose prolotherapy.

Target Category	Representative Evidence	Recent Advance	Interpretation	Key Limitation
Sports tendinopathy	Systematic review of randomized controlled trials	Condition-specific synthesis	Condition-specific comparative evidence; protocol heterogeneity remains	Variation in technique and rehabilitation
Lateral elbow	Meta-analysis and randomized trials	Dose comparison and long-term multiarm data	Limited comparative evidence; no clear universal concentration	Comparator and injection-pattern variation
Rotator cuff/supraspinatus	Systematic review and randomized controlled trials	Targeted ultrasound-guided protocols	Limited comparative evidence with target and follow-up variability	Target location and structural phenotype vary
Plantar fascia	Meta-analyses and randomized controlled trials	Comparisons with steroid and extracorporeal shock wave therapy	Condition-specific evidence is expanding	Durability and protocol standardization
Knee osteoarthritis/periarticular pain	Meta-analyses and recent randomized controlled trials	Concentration and target comparisons	Mixed comparative evidence; non-uniform protocols	Whole-joint phenotyping and causal attribution
Capsular/enthesis/bursa	Site-specific trials and cohort	Expansion beyond tendon and joint cavity	Limited condition-specific evidence	Small samples and limited replication
TMJ	Meta-analyses, randomized and pilot studies	Guidance and comparator studies	Growing but heterogeneous comparative evidence	Diagnostic and procedural inconsistency
Novel anatomical targets	Cadaveric/technical studies and cases	Anatomical pathway development	Anatomical/technical/case-based evidence	Independent clinical validation absent

Note: TMJ, temporomandibular joint. Supporting references are cited in the corresponding Sections in the main text.

**Table 4:** Priority questions and minimum reporting elements.

Research Domain	Priority Question	Minimum Reporting	Preferred Design
Mechanism	How much benefit is attributable to separation, volume, dextrose, needle effect, or contextual care?	Injectate, volume, target plane, observed spread, co-interventions	Factorial or mechanism-oriented randomized trial
Patient selection	Which clinical and imaging phenotypes predict response?	Diagnostic criteria, symptom reproduction, ultrasound findings, electrodiagnostic/imaging data	Prospective phenotype-stratified cohort or trial
Dose and concentration	What volume is required for hydrodissection, and what dextrose concentration is optimal for each connective-tissue target?	Concentration, total volume, injection points, session number	Dose-ranging trial
Procedural endpoint	Do changes in gliding, plane opening, or cross-sectional area predict outcome?	Standardized dynamic maneuver and imaging acquisition	Prospective validation study
Emerging targets	Are novel anatomical pathways safe and clinically effective?	Cadaveric validation, neurovascular safety, independent replication	Stepwise feasibility-to-comparative program
Rehabilitation	How do loading, mobility, and activity modification interact with injection effects?	Exercise type, dose, adherence, activity restriction	Pragmatic multimodal trial
Safety	What are site-specific adverse events and delayed complications?	Standardized adverse-event definitions and follow-up	Registry or adequately powered prospective cohort

Note: The listed questions and preferred designs represent research priorities rather than formal clinical recommendations.

## Data Availability

No new data were created or analyzed in this study. Data sharing is not applicable to this article.

## Supplementary Materials

The following supporting information can be downloaded at https://www.mdpi.com/article/10.3390/life16091438/s1. Table S1: Database-Specific Search Strategies. Table S2: Characteristics of Principal Comparative Clinical Studies Informing the Narrative Synthesis.

## Abbreviations

CTS, carpal tunnel syndrome; D5W, 5% dextrose in water; TMJ, temporomandibular joint.
