# Supplementary material for: Recent Advances in Ultrasound-Guided Hydrodissection and Dextrose Prolotherapy for Musculoskeletal Pain: A Target-Based State-of-the-Art Narrative Review
Source: Life (Basel). 2026 Aug 28;16(9):1438. doi: 10.3390/life16091438 (PMC13608313; doi:10.3390/life16091438)
Supplement: Supplementary file 1 [file life-16-01438-s001.zip › life-4500409-supplementary.pdf]

Supplementary Table S1. Database-Specific Search Strategies

Review period: 1 January 2021 to 29 July 2026; selected pre-2021 landmark publications were identified by backward citation tracking.

Database	Search domain	Search strategy	Limits/notes
PubMed	Hydrodissection	(<br>hydrodissection[Title/Abstract]<br>OR "hydro-dissection"[Title/Abstract]<br>OR "hydro dissection"[Title/Abstract]<br>)<br>AND<br>(<br>ultrasound[Title/Abstract]<br>OR ultrasonograph*[Title/Abstract]<br>OR sonograph*[Title/Abstract]<br>OR "Ultrasonography, Interventional"[Mesh]<br>)<br>AND<br>(<br>perineural[Title/Abstract]<br>OR nerve*[Title/Abstract]<br>OR neural[Title/Abstract]<br>OR neuropath*[Title/Abstract]<br>OR entrapment[Title/Abstract]<br>OR fascia*[Title/Abstract]<br>OR interfascial[Title/Abstract]<br>OR peritendinous[Title/Abstract]<br>OR tendon*[Title/Abstract]<br>OR musculoskeletal[Title/Abstract]<br>)	English; 1 Jan 2021–29 Jul 2026
PubMed	Dextrose prolotherapy	(<br>"Prolotherapy"[Mesh]<br>OR prolotherap*[Title/Abstract]<br>OR "dextrose prolotherapy"[Title/Abstract]<br>OR "glucose prolotherapy"[Title/Abstract]<br>OR "hypertonic dextrose injection"[Title/Abstract]<br>OR "hypertonic glucose injection"[Title/Abstract]<br>OR "dextrose injection"[Title/Abstract]<br>)<br>AND<br>(<br>musculoskeletal[Title/Abstract]<br>OR tendon*[Title/Abstract]<br>OR tendinopath*[Title/Abstract]<br>OR tendinosis[Title/Abstract]<br>OR ligament*[Title/Abstract]<br>OR enthesis[Title/Abstract]<br>OR enthesopath*[Title/Abstract]<br>OR capsul*[Title/Abstract]<br>OR joint*[Title/Abstract]<br>OR fascia*[Title/Abstract]<br>)	English; 1 Jan 2021–29 Jul 2026
PubMed	Perineural dextrose/mechanistic overlap	(<br>dextrose[Title/Abstract]<br>OR "5% dextrose"[Title/Abstract]<br>OR D5W[Title/Abstract]<br>OR "glucose water"[Title/Abstract]<br>)<br>AND<br>(<br>perineural[Title/Abstract]<br>OR hydrodissection[Title/Abstract]<br>OR nerve[Title/Abstract]<br>OR neural[Title/Abstract]<br>)<br>AND<br>(<br>inject*[Title/Abstract]<br>OR treatment[Title/Abstract]<br>OR therapy[Title/Abstract]<br>)	English; 1 Jan 2021–29 Jul 2026
Embase via Ovid	Hydrodissection	(<br>hydrodissection	English; 1 Jan 2021–29 Jul 2026; no full-text-availability filter

Database	Search domain	Search strategy	Limits/notes
		OR hydro-dissection<br>OR (hydro adj1 dissection)<br>).ti,ab,kw.<br>AND<br>(<br>ultrasound<br>OR ultrasonograph*<br>OR sonograph*<br>).ti,ab,kw.<br>AND<br>(<br>perineural<br>OR nerve*<br>OR neural<br>OR neuropath*<br>OR entrapment<br>OR fascia*<br>OR interfascial<br>OR peritendinous<br>OR tendon*<br>OR musculoskeletal<br>).ti,ab,kw.	
Embase via Ovid	Dextrose prolotherapy	(<br>prolotherap*<br>OR (dextrose adj3 prolotherap*)<br>OR (glucose adj3 prolotherap*)<br>OR (hypertonic adj3 dextrose adj3 inject*)<br>OR (hypertonic adj3 glucose adj3 inject*)<br>OR ((dextrose OR glucose) adj3 inject*)<br>).ti,ab,kw.<br>AND<br>(<br>tendon*<br>OR tendinopath*<br>OR tendinosis<br>OR ligament*<br>OR enthesis<br>OR enthesopath*<br>OR capsul*<br>OR musculoskeletal<br>OR joint*<br>OR fascia*<br>).ti,ab,kw.	English; 1 Jan 2021–29 Jul 2026; no full-text-availability filter
Embase via Ovid	Perineural dextrose/mechanistic overlap	(<br>((dextrose OR "5% dextrose" OR D5W OR "glucose water") adj5<br>(perineural OR hydrodissection))<br>OR<br>((dextrose OR "5% dextrose" OR D5W) adj5<br>(nerve OR neural) adj5 inject*)<br>).ti,ab,kw.	English; 1 Jan 2021–29 Jul 2026; no full-text-availability filter

Screening and selection note: PubMed yielded 635 records and Embase yielded 1,411 records across the three search domains. After within-database duplicate handling and application of the English-language limit, 590 PubMed and 1,194 Embase records remained. Initial cross-database deduplication yielded 1,662 records for title/abstract screening. One author (Y.Y.) performed title/abstract screening, and a second author reviewed the final publication selection. Final inclusion was purposive for a state-of-the-art narrative synthesis rather than a PRISMA-style exhaustive systematic-review cohort.

Supplementary Table S2. Characteristics of Principal Comparative Clinical Studies Informing the Narrative Synthesis

This table provides study-level context for principal comparative clinical studies and is not an exhaustive systematic-review evidence table.

Study	Design / N	Condition	Intervention / comparator	Follow-up	Principal outcomes	Representative finding	Safety / major limitation
Eyvaz et al., 2025 [16]	Double-blind RCT; N=80	Mild–moderate CTS	US-guided median-nerve HD; 5 or 10 mL D5W vs 5 or 10 mL saline; one session	12 weeks	VAS, BCTQ, grip/pinch, CSA, electrophysiology	10 mL D5W showed greater pain and CSA improvement than saline groups by 12 weeks	No universal optimal volume established; protocol-specific finding
Huang et al., 2024 [17]	Single-blind RCT; N=24	Moderate CTS	US-guided HD with 10 vs 5 mL saline; one session	24 weeks	BCTQ symptom/function scores	At 24 weeks, 10 mL vs 5 mL: BCTQ symptom severity median change -0.8 vs -0.5 (P=0.024) and functional status mean change -0.8 vs -0.5 (P=0.011).	Small sample; saline injectate; single technique
Shi et al., 2024 [35]	Double-blind RCT; N=56	Meralgia paresthetica	US-guided 10 mL D5W HD vs corticosteroid HD; one session	6 months	Pain/paresthesia, quality of life, response, adverse effects	Both groups improved; D5W favored selected outcomes at 4–6 months	Single condition/site; independent replication limited
Mansiz-Kaplan et al., 2022 [36]	Double-blind RCT; N=40	Ulnar neuropathy at elbow	US-guided multi-level perineural D5W vs saline; 5 mL total	12 weeks	Pain, disability, conduction, CSA	D5W improved several clinical/electrophysiologic outcomes versus saline	Perineural injection; tissue-plane separation not established as defining endpoint
Lin et al., 2022 [25]	Double-blind placebo-controlled RCT; N=57	Chronic supraspinatus tendinosis	US-guided 20% dextrose vs saline	12 weeks	VAS, SPADI, ROM, ultrasound morphology	At 2 weeks, within-group VAS mean difference -2.1 (95% CI -2.7 to -1.4) and SPADI -11.6 (95% CI -16.5 to -6.7); effect was not sustained to week 6.	Target- and time-specific effect; modest sample
Raissi et al., 2023 [45]	Randomized double-blind trial	Chronic plantar fasciitis	US-guided 20% dextrose vs corticosteroid; single injection	12 weeks	Pain, function, fascia thickness	Corticosteroid favored early outcomes; groups were more similar by later follow-up	Time-dependent comparator effect; no injection-related adverse events reported
Ciftci et al., 2023 [49]	Double-blind RCT; N=60	Chronic lateral epicondylitis	US-guided 5% vs 15% dextrose vs saline; 0, 3, 6 weeks	12 weeks	Pain, grip, pressure-pain threshold, disability	Both dextrose groups improved; selected outcomes favored 15%	Single trial does not establish a universal concentration
Teymouri et al., 2025 [54]	Double-blind RCT; 50 completers	Knee OA	US-guided 25% dextrose vs intra-articular saline; one session	8 weeks	Oxford Knee Score, VAS, WOMAC, ROM, adverse events	Both groups improved; between-group differences were not significant for key outcomes at 8 weeks	Short follow-up; transient injection-site pain/ecchymosis

Study	Design / N	Condition	Intervention / comparator	Follow-up	Principal outcomes	Representative finding	Safety / major limitation
Babaei-Ghazani et al., 2024 [55]	Randomized multicenter trial	Pes anserine bursitis	US-guided prolotherapy vs oxygen–ozone vs corticosteroid	Study-specific	Pain/function	Direct condition-specific comparison across three injectates	Comparator complexity; site-specific generalizability
Lin et al., 2023 [56]	Double-blind RCT	Chronic subacromial bursitis	Hypertonic dextrose vs steroid injection	Study-specific	Pain/function	Direct condition-specific comparison	Single condition and protocol
Alhaj Kheder et al., 2024 [59]	Randomized clinical trial	TMJ internal derangement	Ultrasound-guided vs non-guided prolotherapy	Study-specific	TMJ clinical outcomes	Directly evaluates the role of ultrasound guidance	Diagnostic and procedural heterogeneity across the field

Abbreviations: BCTQ, Boston Carpal Tunnel Syndrome Questionnaire; CI, confidence interval; CSA, cross-sectional area; CTS, carpal tunnel syndrome; D5W, 5% dextrose in water; HD, hydrodissection; OA, osteoarthritis; RCT, randomized controlled trial; ROM, range of motion; SPADI, Shoulder Pain and Disability Index; TMJ, temporomandibular joint; VAS, visual analog scale.
